# Lean Body Mass Associated with Upper Body Strength in Healthy Older Adults While Higher Body Fat Limits Lower Extremity Performance and Endurance

**DOI:** 10.3390/nu7095327

**Published:** 2015-08-26

**Authors:** Karen Charlton, Marijka Batterham, Kelly Langford, Jenna Lateo, Erin Brock, Karen Walton, Philippa Lyons-Wall, Katie Eisenhauer, Nick Green, Cameron McLean

**Affiliations:** 1 School of Medicine and Statistical Consulting Centre, University of Wollongong, New South Wales 2522, Australia; E-Mails: kl704@uowmail.edu.au (K.L.); jennalateo@gmail.com (J.L.); eeb786@uowmail.edu.au (E.B.); kwalton@uow.edu.au (K.W.); katie_e@balancepodiatry.com.au (K.E.); Nick.Green@bupa.com.au (N.G.); camo.mclean@bigpond.com (C.M.); 2 Statistical Consulting Services, National Institute of Applied Statistics Research Australia, University of Wollongong, Wollongong, New South Wales 2522, Australia; E-Mail: marijka@uow.edu.au; 3 School of Exercise and Health Sciences, Edith Cowan University, Joondalup WA 6027, Australia; E-Mail: p.lyons-wall@ecu.edu.au

**Keywords:** older people, body composition, physical function, upper body strength, lean body mass, protein

## Abstract

Impaired strength adversely influences an older person’s ability to perform activities of daily living. A cross-sectional study of 117 independently living men and women (age = 73.4 ± 9.4 year; body mass index (BMI) = 27.6 ± 4.8 kg/m^2^) aimed to assess the association between body composition and: (1) upper body strength (handgrip strength, HGS); (2) lower extremity performance (timed up and go (TUG) and sit to stand test (STS)); and (3) endurance (6-minute walk (SMWT). Body composition (% fat; lean body mass (LBM)) was assessed using bioelectrical impedance. Habitual physical activity was measured using the Minnesota Leisure Time Physical Activity Questionnaire (MLTPA) and dietary macronutrient intake, assessed using 24 h recalls and 3-day food records. Regression analyses included the covariates, protein intake (g/kg), MLTPA, age and sex. For natural logarithm (Ln) of right HGS, LBM (*p* < 0.001) and % body fat (*p* < 0.005) were significant (*r*^2^ = 46.5%; *p* < 0.000). For left LnHGS, LBM (*p* < 0.000), age (*p* = 0.036), protein intake (*p* = 0.015) and LnMLTPA (*p* = 0.015) were significant (*r*^2^ = 0.535; *p* < 0.000). For SMW, % body fat, age and LnMLTPA were significant (*r*^2^ = 0.346; *p* < 0.000). For STS, % body fat and age were significant (*r*^2^ = 0.251; *p* < 0.000). LBM is a strong predictor of upper body strength while higher % body fat and lower physical activity are associated with poorer outcomes on tests of lower extremity performance.

## 1. Introduction

Ageing is characterised by changes in body composition, including decreased muscle mass, or sarcopenia, and an accompanying increase in fat mass [1,2,3,4,5,6]. Sarcopenia, defined as a skeletal muscle of less than two standard deviations (SD) of the mean for young persons, is associated with loss of strength and function and has been linked to a 3–4 fold increased risk of disabilities, falls, functional impairment, loss of independence and a decreased quality of life [2,7,8,9,10]. The proportion of people estimated to be affected by sarcopenia ranges between 5 to 13% in persons aged 60 to 70 years and 11 to 55% in persons aged 80 years and above [6]. In 2000, the estimated direct health care expenses related to sarcopenia was estimated to be $18.5 billion in the United States, placing an immense strain on the health care system [11,12].

Measurements of body composition are strong determinants of functionality and mortality in older adults [13]. Adiposity, measured by a percentage of body fat, has been shown to exacerbate the age-related decline in physical function [5,14]. It is estimated that after 20 years of age, fat-free mass progressively decreases, while fat mass increases, with a maximal fat mass reached around 60 years of age [5].

The healthy weight range for adults over 65 years of age is a body mass index (BMI) between 22 and 27 kg/m^2^, whereas a BMI exceeding 30 kg/m^2^ is associated with increased functional disability and reduced physical performance, in particular mobility, when compared to lean adults of the same age [5,15]. Conversely, significant weight loss and a low BMI (BMI < 22 kg/m^2^) is also associated with a decline in physical function and physical performance in the aging population including low appendicular muscle mass, suboptimal grip strength and slow walking speeds [14,16]. An increase in mortality risk in older adults with a BMI < 23 kg/m^2^, but not in the overweight group with a BMI > 27 kg/m^2^, was also shown in a recent meta-analysis [17].

BMI is commonly used to assess body composition as it is easily measured and does not require costly equipment. However, it should be used with caution as it is unable to distinguish between quantities of fat mass and muscle mass and for any given value of BMI, the ratio of fat mass to muscle mass may vary [5,14]. Therefore, measurements of body composition in terms of muscle mass and muscle strength, are thought to provide a more accurate reflection of physical function and ability [14].

The loss of muscle mass is one of the prominent changes in body composition that occurs with aging and is associated with a deterioration in physical function and performance [15,18]. However, while losses in muscle mass have been shown to impact physical performance [19], muscle strength has been found to decline more rapidly with age than muscle mass and is more strongly associated with physical dysfunction, functional limitations and mortality [7,13].

Muscle strength and endurance are essential for performing activities of daily living and participating in physical activity. Strength has been shown to be diminished in persons whom are not regularly active and a loss of strength predicts an increased risk of physical dysfunction and disability in older adults [16]. This highlights the importance of maintaining a physically active lifestyle in slowing the progression of muscle strength deterioration that occurs with age. Being physically active can also contribute to a reduced risk of developing obesity, hypertension, diabetes mellitus, heart disease and osteoporosis [4]. As it is difficult to regain muscle tissue and strength once it has been lost, prevention of this process becomes paramount for older people to maintain independence with activities of daily living and a higher quality of life [7,13].

Given the increase in functional impairment and disability in the growing older population, it is important to understand how body composition may impact upper body strength and physical function in older adults before they become frail. We hypothesize that in healthy independently-living older adults, a lower lean body mass will be associated with lower upper body strength and poorer outcomes in tests of endurance and lower extremity performance.

## 2. Experimental Section

### 2.1. Participants

One hundred and seventeen men and women were recruited from independent living community-dwellings across the lllawarra and Southern Highlands’ area of New South Wales, Australia, between 2010 and 2014. Recruitment strategies included letter box drops, distribution of flyers in common rooms and information sessions held at residential facilities. Participants were required to be at least 55 years old and have a good understanding of English. Ineligibility criteria included uncontrolled hypertension, unstable type 1 diabetes, having a pacemaker, severe dementia or dysphasia, a physical disability that limits walking or dependency on others for activities of daily living, cognitive impairment and/or food allergies.

The study was approved by the University of Wollongong Human Research Ethics Committee (HE15/178) and written consent was obtained from all participants prior to taking part in this study.

### 2.2. Anthropometric Measures and Body Composition

Height was measured to the nearest 0.1 cm using a stadiometer (SECA 217 1 mm graduation) and weight with floor scales (SECA 874 +/− 100 g). Body composition was measured using either bioelectrical impedance analysis (BIA) (Tanita Segmental Body Composition Analyser, Model BC-418, Tanita Corporation of America Inc, IL, USA) (*n* = 66) or Tanita foot-to-foot stand on scales (UM-019) (*n* = 43). Both these models use the same prediction equations to estimate body fat mass, which include gender, age and height in the algorithm, along with weight and impedance data. Fat free mass was calculated as (total body mass (kg)–fat mass (kg)). Body fat was expressed as a percentage of total body mass. Body mass index was calculated as weight/height squared (kg/m^2^). A validation study was conducted in a sub-sample of the first 51 participants recruited to the study in order to assess how the Tanita BIA-418 segmental body analyser compared against measures obtained using a dual-energy X-ray absorptiometer (DXA) scan, performed using a Norland XR46 bone densitometer machine, (Norland at Swissray, Fort Atkinson, WI, USA).

### 2.3. Muscle Strength, Lower Body Extremity Performance, and Endurance

Upper body strength (kg) was measured using a hand dynamometer (Model Jamar Plus, Sammsons Preston Rolyan, Bolingbrook, IL, USA). Participants were asked to sit in a chair, feet flat on the floor with their dominant arm adducted with their elbow at a 90 degree angle. On the count of three, they were instructed to exert maximal force for 10 s before releasing. Hand-held dynamometry has good test-retest reliability and concurrent validity in older community dwelling persons [20].

The 30-s sit-to-stand (STS) test was used to measure lower body extremity performance and assessed the participant’s ability to stand up from a chair without the use of their arms. The participants started from a seated position in a chair with their arms folded across their chest and were instructed to stand fully upright and return to the seated position as many times as they could manage comfortably in 30 s. The final score was the number of stands completed in 30 s. The 30-s sit-to-stand test has demonstrated good test-retest reliability and provides a valid indication of lower body strength in generally active, community-dwelling older adults [21].

The timed up and go test was used to measure both lower extremity performance and dynamic balance performing three common functional activities such as standing up from a chair and walking and turning through the evaluation of mobility [22]. For this test, participants were required to stand up from the chair, walk 3 m at a comfortable speed, turn around and walk back to the chair and sit down. The final score was the time taken to complete this task. The timed up and go test provides a valid indication of lower extremity functional ability in generally active, community-dwelling older adults and has good test-retest reliability [22].

The 6-min walk test was used to measure endurance. This test required the participants to walk a 10 m course at a comfortable speed for 6 min using walking frames or aides normally used, if required for daily living. The number of laps completed during the 6 min were tallied to calculate the total distance in meters walked. The 6 min walk test has good test-retest reliability and provides a clinically valid measure of endurance in an older population [23].

### 2.4. Nutritional Status and Dietary Intake

Dietary intake was measured by asking participants to keep a food record (*n* = 47) for 3 consecutive days (one weekend day and two weekdays) or from a 24 h recall (*n* = 69). These were analysed using FoodWorks using NUTTAB 2010, Ausfoods 2012 and Ausbrands 2012 database (Xyris Software, Highgate Hill, GLD, Australia, Version 6, 2009). A random cross check of 10% of the food records was conducted by two fieldworkers for quality control and to check accuracy.

The Mini Nutritional Assessment (MNA^®^) [24], a validated questionnaire designed for use in persons aged 65 years and above, was conducted by a trained researcher to determine overall nutritional status for the purpose of describing the sample, with regard to generalizability of the results. A score > 23.5 out of a possible 30 indicates that an individual is well-nourished; a score between 17 and 23.5 indicates that the individual is at risk of malnutrition, and a score < 17 indicates that is individual is malnourished.

### 2.5. Reported Physical Activity

The Minnesota leisure time physical activity questionnaire (MLTPA) was used to assess habitual physical activity over the last year [25]. An intensity measure was allocated to 64 activities, ranging from 2.0 for light to 6.0 for heavy intensity. Participants were required to record their participation in various activities over the last 12 months, as well as the months involved, the frequency each month and the time per session. Results were reported as total Activity Metabolic Index (AMI)/week calculated as activity intensity code × duration × times per month × months per year/52.

### 2.6. Data Analysis

Statistical Package for the Social Sciences (SPSS) was used for statistical analysis (SPSS version 21.0, Chicago, IL, USA). Independent *t*-testing was used to compare mean values for male and female data. Bivariate correlations were performed between independent variables and handgrip strength, physical function tests, and physical activity. Multivariate regression models were conducted for the following dependent variables: Right hand grip strength, left hand grip strength, sit to stand, timed up and go and six minute walk test, a backward stepwise regression model was also used for the timed up and go test due to the smaller sample size. Independent variables included in the models were: BMI or % body fat, fat free mass (kg), protein intake (g) per kg body weight, physical activity (MLTPA), age and sex. Skewed variables were log transformed (logarithm (Ln) hand grip strength (right and left) and Ln physical activity) and all models were compared with and without transformation. Where transformed variables gave a better model fit that accounted for more variance, these models are reported. Significance was accepted at *p* < 0.05.

## 3. Results

One hundred and seventeen participants, aged 55 to 90 years of age, volunteered to participate in this study. Table 1 includes a description of physical characteristics and upper and lower body strength and endurance performance results of the participants, as well as their current nutritional status and key nutrient intakes.

The average age of participants was 73.4 ± 9.4 years with no significant difference between genders. Men weighed significantly less than women (71.7 ± 17.9 kg, 75.1 ± 10.7 kg, respectively; *p* = 0.019), but had comparable percentage body fat (men 34.8 ± 7.5%, women 34.6 ± 8.6%, respectively; *p* = 0.908). Mean BMI for this study population (*n* = 116) fell in the overweight range (27.6 ± 4.8 kg/m^2^). Fifteen participants were classified as underweight with a BMI of less than 22 kg/m^2^, while 45 participants were found to be in the healthy weight range (BMI between 22 and 27 kg/m^2^). Mean MNA^®^ score for the study population was 27.2 ± 2.5 (“Normal Nutritional Status”). Sixteen out of 117 (13.7%) were “At risk of malnutrition” with an MNA^®^ score between 17 and 23.5 and none were considered malnourished.

Women had significantly greater participation in physical activity than men (median (Inter Quartile Range; IQR) = 1776 (2077) AMI/week *vs*. 1316 (1711) AMI/week, respectively, *p* = 0.050), however no significant differences were found between genders for measures of hand grip strength, lower extremity performance (sit-to-stand, timed up and go) or functional performance (6-min walk test).

**Table 1:** Characteristics of study participants: Medians and interquartile range (25th and 75th percentile).

Characteristics	Total	Men	Women	*p* Value
	(*n* = 117)	(*n* = 65)	(*n* = 52)	
Age (years)	74.0 (13.0)	73.0 (10.1) #	73.9 (8.5) #	0.612
Height (m)	162.3 (8.5) #	161.4 (8.0) #	163.5 (9.0) #	0.199
Weight (kg)	71.9 (1.3) #	71.7 (17.9)	75.1 (10.7) #	0.019 *
BMI (kg/m^2^) †	27.10 (5.9)	25.5 6.8)	27.9 (5.2)	0.045 *
Body Fat %	34.7 (8.0) #	34.8 (7.5) #	34.6 (8.6) #	0.908
Fat free mass (kg)	46.7 (9.1)	44.9 (9.2)	48.9 (8.5)	0.021
MNA Score ‡	28.0 (3.0)	27.5 (3.0)	28.0 (2.5)	0.105
Grip Strength RH (kg) §	23.95 (10.0)	23.72 (8.7)	24.1 (17.3)	0.231
Grip Strength LH (kg) §	22.4 (12.3)	21.8 (8.0) #	23.2 (16.1)	0.089
6 Minute Walk (SMW) (m)	366.4 (135.4) #	359.8 (152.2) #	373.7 (115.8) #	0.636
Sit-to-stand (STS) (reps) ††	13.0 (6.0)	13.5 (5.4) #	13.3 (5.0)	0.789
Timed Up and Go (TUG) (s) ‡‡	9.6 (4.3)	10.8 (2.9) #	9.0 (4.8)	0.727
Physical Activity (AMI/wk)	1520 (1858)	1316 (1711)	1776 (2077)	0.050 *
Energy (kJ) §§	7287 (2643)	7319 (2529)	7255 (3100)	0.745
Protein (g)	81.3 (36.2)	82.9 (37.2)	81.9 (29.4) #	0.450
Fat (g)	65.3 (41.3)	67.7 (28.8) #	67.0 (26.4)	0.881
Carbohydrate (g)	192.0 (81.9)	195.0 (72.7)	189.1 (93.1)	0.882

** *p* < 0.01, * *p* < 0.05; # Mean (standard deviation (SD)) for normally distributed data; † Healthy BMI for adults aged 65+ year is 22–27 kg/m^2^ [5]; ‡ Mini Nutritional Assessment, with a score greater than 23.5 indicating no risk of malnutrition [24]; § Median maximum hand grip strength for men and women aged 50+ year are 37.9 kg and 31.5 kg, respectively [19]; Recent meta-analysis of six minute walk test results found mean distance covered by men aged over 60 was 524 m and by women 475 m [26]; †† Median sit-to-stand test results for men and women aged 75+ year are 10.9 and 11.9 repetitions, respectively [27]; ‡‡ Median timed up and go results for men and women aged 70+ year are 9.87 and 11.73 s, respectively [28]; §§ The estimated energy requirements for a moderately active man aged 70+ year, weighing 80 kg is 10,000 kJ a day, and for women aged 70+ year , weighing 65 kg is 8000 kJ a day using the Schofield equation [29]; Estimated average protein requirement for men and women aged 70+ year is 65 g and 40 g, respectively [29].

Correlations between body composition, nutritional status, strength and endurance are shown in Table 2. 

**Figure 1:**
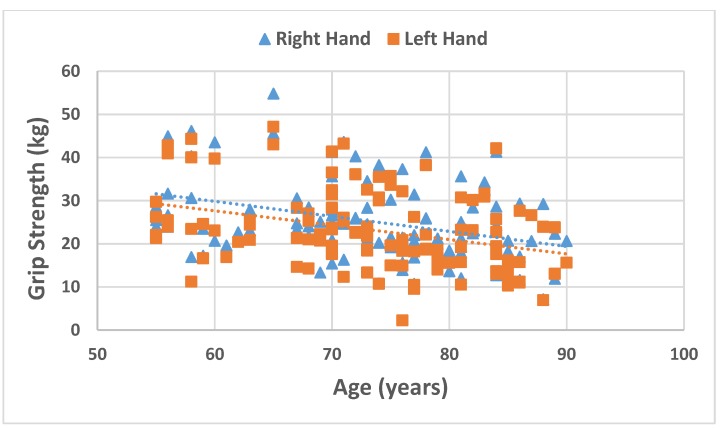
Associations between age and right and left hand grip strength.

Both left and right hand grip strength were inversely associated with age (*r* = −0.344, *p* > 0.001, *r* = −0.365, *p* > 0.001, respectively; Figure 1).

**Table 2:** Bivariate associations between body composition, nutritional status, strength and endurance (Pearson’s correlations, *r*).

		Upper Body Strength		Lower Body Strength		Lower Body Endurance	Physical Activity
		Hand Grip		STS	TUG †	SMW	AMI †
		Right †	Left				
		(Men: *n* = 64; Women: *n* = 52)	(Men: *n* = 65; Women: *n* = 51)	(Men: *n* = 61; Women: *n* = 49)	(Men: *n* = 17; Women: *n* = 31)	(Men: *n* = 44; Women: *n* = 40)	(Men: *n* = 63; Women: *n* = 50)
		***R***	***r***	***r***	***r***	***r***	***R***
**Age**	Men	−0.238	−0.141	−0.492 **	0.155	−0.492 **	−0.427 **
	Women	−0.547 **	−0.620 **	−0.413 **	0.449 *	−0.555 **	−0.143
**Weight (kg)**	Men †	0.213	0.338 **	−0.198	0.452	−0.295	−0.097
	Women	0.318 *	0.313 *	0.131	0.209	0.117	0.105
**BMI (kg/m^2^)**	Men †	−0.069	0.060	−0.268 *	0.386	−0.362 *	−0.030
	Women †	−0.196	−0.211	−0.240	0.185	−0.266	−0.107
**Body fat (%)**	Men	−0.475 **	−0.412 **	−0.073	0.163	−0.143	0.086
	Women	−0.580 **	−0.673 **	−0.569 **	0.268	−0.634 **	0.204
**MNA**	Men †	0.025	0.19	0.390 **	0.379	0.293	0.273 *
	Women	0.123	0.151	0.161	−0.040	0.317 *	0.181
**Dietary intake**							
**Energy (kJ)**	Men †	0.039	0.002	0.043	−0.283	−0.001	−0.034
	Women	0.429 **	0.431 **	0.196	−0.242	0.342 *	0.250
**Protein (g)**	Men †	0.202	0.109	0.088	−0.177	0.063	0.211
	Women	0.186	0.173	0.167	−0.237	0.190	0.199
**Fat (g)**	Men	0.032	−0.064	−0.208	−0.221	−0.158	−0.148
	Women	0.287 *	0.283 *	0.101	−0.106	0.279	0.282 *
**CHO (g)**	Men †	0.163	0.092	0.034	−0.305	−0.063	0.023
	Women	0.429 **	0.460 **	0.192	−0.228	0.279	0.110

† Not normally distributed data; Spearman’s correlation used; ** *p* < 0.01, * *p* < 0.05; Legend: STS: Sit-To-Stand Test; TUG: Timed Up and Go Test; SMW: Six-Minute Walk Test; AMI/week: Activity Metabolic Index; BMI: Body Mass Index; MNA: Mini Nutritional Assessment; CHO: Carbohydrate.

**Figure 2:**
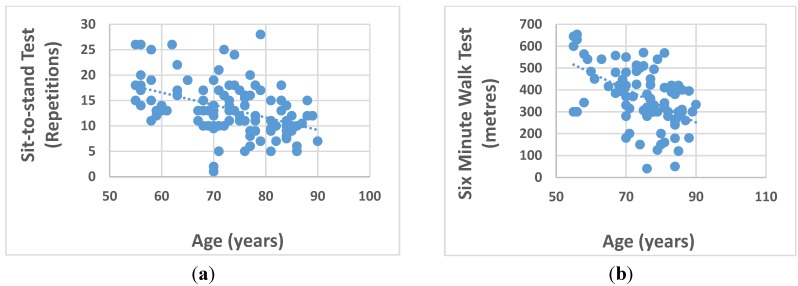
(**a**) Association between sit-to-stand test and age; (**b**) Association between the six minute walk test and age.

Performance on the physical function tests declined with age, as shown in Figure 2 (a. sit to stand test: *r* = −0.463, *p* < 0.01; b. six minute walk test and age: *r* = −0.516, *p* < 0.01).

**Figure 3:**
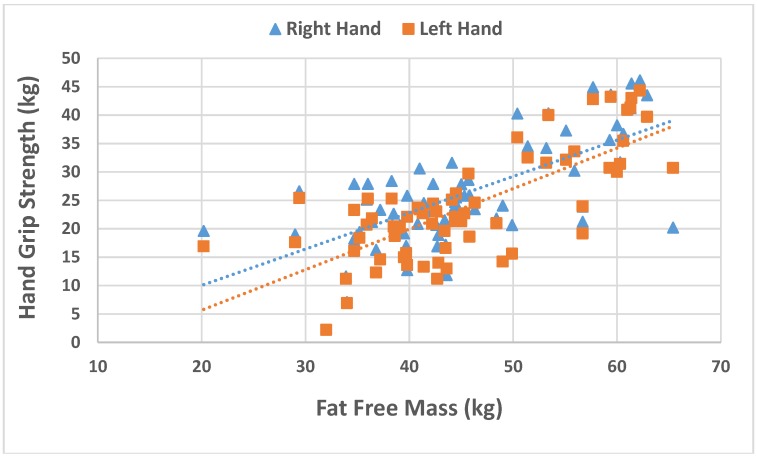
Association between right and left hand grip strength and fat free mass.

A positive association was found between left and right hand grip strength and fat free mass (*r* = 0.710; *p* < 0.001 and *r* = 0.625; *p* < 0.001, respectively), as shown in Figure 3.

In all five of the multiple regression models, BMI was not significant therefore percentage body fat was used instead as this increased the amount of variance explained. Protein intake (g/day) adjusted for body weight (kg) using regression residuals gave a better model fit than protein intake expressed per kg body weight. Results for each of the models are shown in Table 3 and summarized below.

Right hand grip strength (ln transformed): The overall model was statistically significant (F = 15.62, d*f* = 6.95; *p* < 0.000), accounting for 46.5% of the variance. As indicated by the significance of the t statistics and the standardized coefficients lean body mass was the strongest predictor (*t* = 4.99; *p* < 0.001), followed by percentage body fat (*t* = −2.86; *p* <0.005). None of the other variables were significant in the model.

Left hand grip strength (ln transformed): The overall model was statistically significant (F = 20.54, d*f* = 6.96; *p* < 0.000), accounting for 53.5% of the variance. Again, lean body mass was the strongest predictor (*t* = −6.38 *p* < 0.000). Age (*t* = −2.13; *p* = 0.036), adjusted protein intake (*t* = −2.49; *p* = 0.015) and logarithm Minnesota Leisure Time Physical Activity Questionnaire (LnMLTPA) (*t* = 2.64, *p* = 0.015) were also significant. There was a single bivariate outlier in this model who only had a left handgrip measurement recorded; this subject had a standardized residual of −4.3. The model rerun without this subject provided similar results, therefore the analysis that included the subject is reported.

Six minute walk test: The overall model was statistically significant (F = 7.10, d*f* = 6.93; *p* < 0.000), accounting for 34.6% of the variance. Percentage body fat, age and LnMLTPA were the significant predictors in the model.

Sit-to-stand test: The overall model was statistically significant (F = 6.46, d*f* = 6.92; *p* < 0.000), accounting for 25.1% of the variance. Percentage body fat and age were the significant predictors with LnMLTPA having a borderline significance in the model.

Timed Up and Go: This model included only 30 subjects for whom measurements were collected for the test. The model was statistically significant (F = 3.19, d*f* = 6. 30; *p* = 0.015), and accounted for 26.7% of the variance. Age and gender were of borderline significance. Given the reduced sample size, a stepwise model was also run. This model was of borderline significance (*p* = 0.041) and contained LnMLTPA as the only predictor.

Variance inflation factors were calculated for all models and were all below 3.3, thus there was no evidence of multicollinearity between body composition parameters included in the models (e.g., % fat mass and fat-free mass).

Thirty-four participants participated in the DEXA validation sub-study (*n* = 20 women; *n* = 14 men). In women, there were no statistically significant differences between body composition as measured by BIA and DEXA for body fat percentage (36.5% and 34.9% respectively, *p* = 0.169), fat mass (23.9% and 23.4% respectively, *p* = 0.553) and fat free mass (40.5% and 40.2% respectively, *p* = 0.685). In men, differences were found for measures of body composition assessed using BIA and DEXA (body fat percentage (27.2% *vs*. 21.5%, *p* < 0.001); total fat mass (22.1%, *vs*. 18.0%, *p* < 0.001) and fat free mass (58.6% *vs*. 61.0%, *p* = 0.008).

**Table 3:** Multivariate mixed effects regression models for strength and physical function outcomes.

Models	Beta coefficient	Standard Error	*T*	*p*
Right handgrip strength (ln transformed)				
Age	−0.009	0.003	−2.780	0.007
Gender	−0.002	0.057	−0.040	0.968
LnMLTPA	0.036	0.031	1.163	0.248
Regression residuals of protein intake/body weight	0.000	0.001	0.258	0.797
Body fat percentage	−0.011	0.004	−2.859	0.005
Fat free mass	0.017	0.003	4.985	0.000
**Left hand grip strength (ln transformed)**				
Age	−0.008	−0.161	−2.132	0.036
Gender	−0.004	−0.004	−0.062	0.951
LnMLTPA	0.085	0.187	2.464	0.015
Regression residuals of protein intake/body weight	−0.003	−0.174	−2.488	0.015
Body fat percentage	−0.012	−0.211	−2.720	0.008
Fat free mass	0.025	0.505	6.382	0.000
**Six minute walk test**				
Age	−5.256	1.402	−3.750	0.000
Gender	11.240	25.284	0.445	0.658
LnMLTPA	29.885	13.197	2.264	0.027
Regression residuals of protein intake/body weight	−0.099	0.409	−0.243	0.809
Body fat percentage	−5.589	1.691	−3.305	0.002
Fat free mass	−2.194	1.453	−1.510	0.136
**Sit-to-stand test**				
Age	−0.206	0.054	−3.851	0.000
Gender	0.225	0.982	0.229	0.819
LnMLTPA	1.061	0.544	1.952	0.054
Regression residuals of protein intake/body weight	−0.010	0.016	−0.654	0.515
Body fat percentage	−0.192	0.065	−2.941	0.004
Fat free mass	−0.093	0.057	−1.616	0.110
**Models**	**Beta coefficient**	**Standard Error**	***T***	***p***
**Timed Up and Go test**				
Age	0.145	0.074	1.961	0.059
Gender	0.133	1.529	0.087	0.931
LnMLTPA	−0.833	0.423	−1.969	0.058
Regression residuals of protein intake/body weight	−0.010	0.016	−0.617	0.542
Body fat percentage	0.122	0.063	1.934	0.063
Fat free mass	0.160	0.065	2.446	0.021

LnMLTPA: logarithm Minnesota Leisure Time Physical Activity Questionnaire.

## 4. Discussion

This study identified that in a sample of older Australian men and women, body composition differed between genders, however measures of upper body strength, and lower extremity functional performance were similar. Despite being generally healthy, both men and women performed poorly compared to published reference standards for their age in the 6-minute walk test [26] and mean maximum hand grip strength [19], however performed well on the sit-to-stand test [27]. Men performed poorly in the timed up and go test, while women performed well for their age [28]. The population were generally well nourished [24], with most men and women consuming above the recommended intake of protein [29]. Surprisingly, men had lower body weight, BMI and fat-free mass than women in this sample, but this probably reflects the convenient sampling frame.

Both upper body strength and performance on lower extremity functional tests declined with age in this study. Right and left hand grip strength (proxy measure for upper body strength), as well as performance in the timed up and go and sit-to-stand tests (measures of lower extremity function), and performance in the 6-min walk test (indicator of endurance), decreased with age in women. For men, only performance in the sit-to-stand and 6-min. walk tests declined with age. These findings are consistent with previously published literature which highlights a reduction in strength with advancing age that consequently results in physical dysfunction and loss of independence [16,18,30,31]. Older adults that have higher amounts of fat-free mass tend to experience a slower decline in strength with age [30,32]. In a 5 year follow-up of men and women aged 75 years at baseline, fat-free mass at baseline was associated with a slower decline in muscle strength [32]. Interventions that increase fat-free mass have been shown to slow the age-related decline of strength in older adults [33].

Our findings identified that a high percentage of body fat was detrimental to both lower extremity performance and endurance in older people, as has been reported in a systematic review [15]. Previous studies have reported walking performance to be poorest in those older adults who have a BMI that exceeds 30 kg/m^2^ and fat mass, not appendicular muscle mass, to be associated with walking speed after adjusting for BMI [14,34]. In our study, BMI did not predict upper body strength, nor ability to perform in the physical function tests that require lower body strength (timed up and go, six min walk and sit to stand). However, we did find a consistent and inverse association between percentage body fat and both upper body strength and lower extremity performance, as well as endurance. Lean body mass (kg), on the other hand, was a stronger predictor of upper body strength but was not significant in the regression models for the other physical function tests. This suggests that preservation of lean body mass is important for upper body strength, but that the presence of excess body fat impairs activities that involve lower body strength and balance.

The need for an adequate dietary protein intake to prevent loss of lean body mass with age is undisputed. Some of the most promising interventions for prevention of sarcopenia have involved supplementation with protein, considering both quantity and quality (type) [35,36,37]. In the present study, the reason why protein intake was associated with only left, but not right, handgrip strength may be related to habitual use of the right hand for daily tasks, sporting activities and previous work-related physical exertion. Strength in the right hand (dominant arm for most participants) may have thus been more influenced by weight bearing activities using this arm, rather than protein intake. Our findings provide support regarding the importance of physical activity and adequate dietary protein intake for optimal body composition and the maintenance of strength and physical function. Interventions aimed at reducing fat mass have demonstrated improved functional performance, independence and quality of life in older adults [38,39].

There are several limitations to the present study. The cross-sectional design is unable to demonstrate causality. Large cohort studies are needed to demonstrate how body composition affects change in upper and lower body strength, and physical function, with advancing age. The choice of tests to assess performance using the lower body extremities may have introduced systematic bias, in that tests such as the sit-to-stand and timed up and go may present difficulties to older adults because of lack of familiarity rather than reflect poor performance *per se* [6]. The use of an objective measure of lower body strength, such as isokinetic strength, may have improved internal study validity [6]. Further, men and women who performed well in one test were also likely to perform well in the other tests. This suggests that the tests assessed a common underlying mobility, rather than distinct functional abilities [27]. However, it is common to use several test instruments to assess lower extremity performance in older adults [40]. The Iowa Established Populations for Epidemiologic Studies of the Elderly study used a battery of tests that included a balance test, a 4-meter walking speed test, and a timed chair sit-to-stand test, with a higher combined score indicating less physical impairment. The Health, Aging, and Body Composition study (Health ABC) developed a similar Physical Performance Battery (PPB) [41]. The Summary Lower-Extremity Performance Scores (SLEPSs) introduced by Sharkey *et al*. [42] included four categories including static and dynamic balance, usual walking speed, and repeated chair sit-to-stand, again, with a higher score indicating better performance.

Underlying disease conditions such as arthritis are potential confounders that may have affected participants’ ability to perform on the strength and functional tests [15]. Another limitation includes the combination of data collected over a prolonged period of time, with varying methods for some variables such as calculation of body fat percentage and measurement of dietary intake. The use of two different models of bioimpedance analysers warrants particular consideration. Our validation study found good agreement between body fat measures assessed using the multi-segmental model (Tanita BC-418) compared with those assessed using DEXA scans. Published studies have confirmed that the foot-to-foot segmental bioimpedance analyser, as used for the final 43 subjects in our study, provides a relatively accurate estimate of percentage body fat in elderly populations [43]. In addition, the same algorithm equations are used in both Tanita models to estimate body fat mass, and these include incorporation of both gender and age.

The use of two different dietary intake methods (24 h recalls for *n* = 69 and 3 day food record *n* = 47) is acknowledged. While the same method used throughout would be optimal, we feel comfortable with the dietary intake estimates as only the energy and macronutrient data was utilized in the statistical analyzes. Previous researchers have reported less than 10% differences for most nutrients, when comparing these methods [44], while others have reported no significant difference in the protein and carbohydrate intakes when both methods were compared [45].

The strength of the study includes the use of a combination of well validated methods with good demonstrated test-retest reliability to assess both physical function and functional exercise performance in this target group. The MNA^®^ tool used to assess the participants current nutritional status is also a well-known validated method [46] as is the MLTPA that was used to assess habitual physical activity [25].

Many studies of this nature focus on frail or sick older samples. The current study is novel in that it was conducted in older people living independently at home, who were generally healthy. Even in this group, significant associations were found between body composition and performance in physical function tests, as well as upper body strength.

## 5. Conclusions

Lean body mass was the most important predictor of upper body strength, controlling for habitual physical activity and dietary protein intake. Excess fat mass, expressed as a proportion of body weight, was found to be detrimental for upper body strength, as well as lower extremity performance and endurance. Interventions aimed at monitoring and improving the nutritional status and dietary intakes of older adults may prevent the decline in physical function with age and improve independence. Also, interventions aimed at reducing fat mass and increasing fat-free mass may help slow the decline in strength and physical function seen with age. Longitudinal studies would be beneficial to investigate how body composition, strength, functional exercise performance and nutritional status changes with age and to identify factors that can influence and maintain these characteristics over time to help improve the independence and quality of life for the older population.
